# Polyphasic Assessment of Aflatoxin Production Potential in Selected *Aspergilli*

**DOI:** 10.3390/toxins11120692

**Published:** 2019-11-26

**Authors:** Stephen Abiola Akinola, Collins Njie Ateba, Mulunda Mwanza

**Affiliations:** 1 Bacteriophage Therapy and Phage Bio-control Laboratory, Department of Microbiology, Faculty of Natural and Agricultural Sciences, North-West University, Private Bag X2046, Mmabatho 2745, South Africa; akinolastephen3@gmail.com (S.A.A.); collins.ateba@nwu.ac.za (C.N.A.); 2 Center for Animal Health Studies, Faculty of Natural and Agricultural Sciences, North-West University, Private Bag X2046, Mmabatho 2745, South Africa

**Keywords:** aflatoxin, *Aspergilli*, feedlots, PCR, fungi

## Abstract

This study investigated the aflatoxin production potentials of selected fungi using a polyphasic approach. Internally transcribed spacer region of the fungi was amplified using the polymerase chain reaction. Forty-five *Aspergillus* strains were further assessed for aflatoxin production using the conventional methods such as growth on yeast extract sucrose, β-cyclodextrin neutral red desiccated coconut agar (β-CNRDCA); expression of the aflatoxin regulatory genes and the use of both thin-layer chromatography (TLC) and high-performance liquid chromatography (HPLC). A large proportion (82.22%) of the isolates harbored the *Nor*-1 gene while 55.56%, 68.89%, and 80% possessed the *ver*-1, *omt*-A, and *afl*R genes, respectively. All 100% the isolates harbored the *afl*J gene. Twenty-three isolates were positive for aflatoxin production based on the yeast extract sucrose medium (YES) test; ammonium vapor test (51%), yellow pigment production (75.5%), and β-CNRDCA tests; and blue/green fluorescence (57.7%). Based on TLC detection 42.2% produced aflatoxins while in the HPLC, total aflatoxin (AFTOT) production concentrations ranged from 6.77–71,453 µg/g. Detectable aflatoxin B1 (AFB1) concentrations obtained from the HPLC ranged between 3.76 and 70,288 µg/g; 6.77 and 242.50 µg/g for aflatoxin B2 (AFB2); 1.87 and 745.30 µg/g for aflatoxin G1 (AFG1); and 1.67 and 768.52 µg/g for aflatoxin G2 (AFG2). AFTOT contamination levels were higher than European Union tolerable limits (4 µg/kg). The regression coefficient was one (*R^2^* = 1) while significant differences exist in the aflatoxin concentrations of *Aspergillus* (*p* ≤ 0.05). This study reports the potentials of *Aspergillus oryzae* previously known as a non-aflatoxin producer to produce AFG1, AFG2, AFB1, and AFB2 toxins. *Aspergillus* species in feedlots of animals reared for food are capable of producing aflatoxins which could pose hazards to health.

## 1. Introduction

Fungi are normal flora in the soil, hays, silage, and grains which exercise decay activities when conditions are favorable for growth [1]. Fungal growth on food and feed substrates does not culminate directly into the release of toxins that may persist in the medium even after the death of fungal pathogens but can be triggered by stress factors in the environment [2]. Mycotoxins are secondary metabolites produced by fungi in food and feed substrates [3]. Mycotoxin contamination could emanate either from the farm or during harvest and post-harvest stages along the food value chains [2]. Mycotoxin production in food and animal feed is influenced by the type of colonizing fungi, climatic conditions, environmental factors such as pH, type of food, or feed substrate, and types of agronomic practices employed in a specific locality [4,5]. Mycotoxins have thus been detected in different food substrates that include; sorghum, millet, maize, rice, wheat, peanuts, sunflower, soybean, turmeric, ginger, black pepper, almonds, walnuts, coconut, and animal feeds [6,7]. When consumed by humans or animals, mycotoxins can pose serious health hazards ranging from acute to chronic toxicity. Specific complications resulting from the consumption of food or feeds contaminated with mycotoxins includes; liver damage, hepatocarcinoma, hepatitis, cirrhosis, and DNA mutations [8,9]. Aflatoxins, fumonisins, trichothecenes, ochratoxins, deoxynivalenol, and zearalenone are among the list of fungal toxins that exist, of which aflatoxin is the most studied across the world [8]. Aflatoxicosis is a disease condition that results from direct or indirect exposure to aflatoxin through food or animals and their related food products [10].

Aflatoxins are a group of mycotoxins produced by the *Aspergillus* group of fungi [2]. Aflatoxins are long-lived as they are cannot be destroyed by heat treatment during the processing of agricultural commodities [4]. Aflatoxins have carcinogenic, mutagenic, and teratogenic effects in hosts [11] and this makes them the most widely researched fungal toxins globally [12]. Aflatoxins are difuranocoumaric compounds [13] produced along the polyketide pathway. The European Commission 1525/98 regulations [14] as well as the World Health Organization (WHO) and the Food and Agricultural Organization (FAO) standards stipulate that the maximum tolerable limits of aflatoxins in food products are 4–20 ng/g for total aflatoxins, 2–5 ng/g for aflatoxin B1 (AFB1), and 0.05 ng/g for the metabolizing form of aflatoxin (AFM1) [13]. In addition, the concentrations 0.05–10 µg/kg are the recommended limits in baby foods while 5 µg/kg is the maximum limit in animal feed as described by Codex Alimentarius Commission [15]. Although these standard limits may vary between countries, in most countries, the maximum limits for AFB1 in foods are within the range of 0–20 μg/kg [16]. Despite these recommended limits, the rate of aflatoxin contaminations in food and animal feed has risen dramatically and has become a menace to food safety, food security, and socioeconomic livelihood in both developed and developing countries [17]. In light of this, most developed nations and developing countries, such as South Africa, have established legislative standards to assist in the regulation of aflatoxins in both food and feed [18]. Aflatoxins are produced by some toxigenic strains belonging to the Flavi group in the genus *Aspergillus* [19]. Out of the over 180 species within this genus, *Aspergillus flavus*, *Aspergillus parasiticus*, and *Aspergillus nomius* have been reported as the major producers of aflatoxins in agricultural commodities [20]. In recent times, more fungal strains have been implicated in aflatoxin production. This includes; *Aspergillus oryzae*, *A. bombycis*, *A. ochraceoroseus*, *A. pseudotamarii*, *A. tamarii*, *A. parvisclerotigenus*, *A. rambellii*, *A. nidulans*, *A. niger*, *A. arachidicola*, and *A. minisclerotigenes* sp. Nov, and some newly emerging strains; *Emericella venezualensis* and *E. astellata* from the genera Emericella [21,22,23].

Out of the 20 known aflatoxins produced by *Aspergillus* strains, AFB1, aflatoxin B2(AFB2), aflatoxin G1 (AFG1), aflatoxin G2 (AFG2), and the metabolizing forms of aflatoxin (AFM1 and AFM2) are the most common form of mycotoxins in food that are associated with mycotoxicosis [24]. Previous reports have documented the presence of aflatoxins in food and animal feed components in several countries [4,20,21,24]. For instance, in developing countries such as Nigeria, South Africa, China, and Zimbabwe, aflatoxins have been detected in maize, peanuts, Bambara nuts, beans, spices, cashew nuts, rice, milk, and corn-starch while in Argentina, soybean and maize are the most implicated food products [25]. Also, in the United States of America, Portugal, the United Kingdom, and Sweden aflatoxin contaminations have been reported in almonds, spices, and Brazil nuts [4,20,21,24]. Despite the increasing awareness on the occurrence of aflatoxin in food and feeds, the proliferation of toxigenic fungal strains has continued to increase, thus creating opportunities for previously non-toxigenic strains to acquire determinants that afford them the potential to produce aflatoxins. In addition, the difficulty in clearly differentiating aflatoxin producing fungi from the non-producers amplifies the need for this study. In this study, we therefore investigated the potential of *Aspergilli* colonizing feedlots of animals reared for food production in Mafikeng, South Africa to produce aflatoxins using a polyphasic approach.

## 2. Results and Discussion

### 2.1. Characterization of Selected Aspergilli

**Figure 1:**
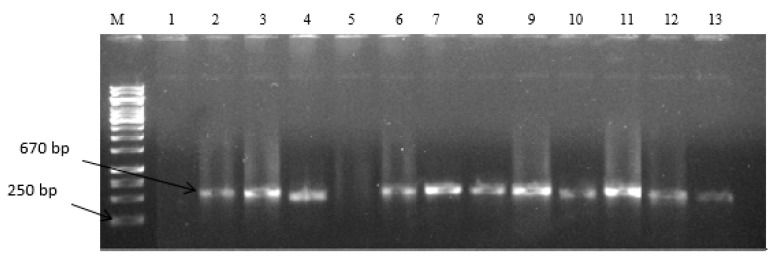
Gel electrophoresis patterns for the expression of ITS1 and ITS4 regions at 670 bp in representative fungi isolated from feedlots of animals reared for food production. Lane M = DNA marker (1 kb); Lane 1 to 13 = positive amplification; Lane 1, and 5 = Negative amplification; and Lane 1 = No template (Internal control), Lane 2 = *Aspergillus flavus ATCC* 259622^TM^ (Positive control).

**Figure 2:**
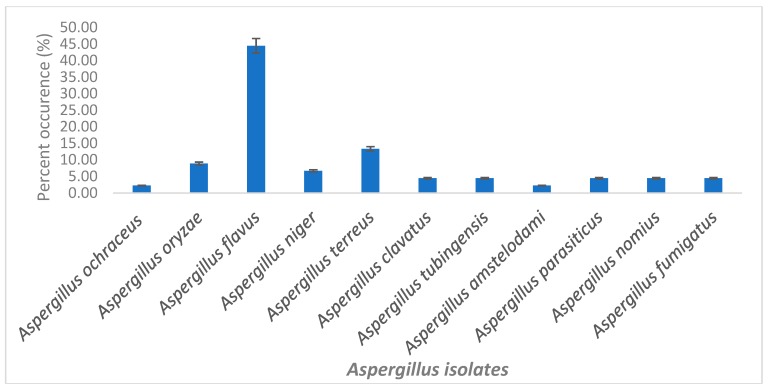
Percent diversity of selected fungi from feedlots of animals kept for food production.

The morphological characteristics of selected *Aspergilli* isolates are presented in Table 1. The pigment variation observed on media plates of isolates ranged from light green, deep green to blue-green, and black pigments. Mycelia were well developed and the identification based on molecular characterization confirms their identity as belonging to the Flavi family. A total of 45 representative fungi isolates had amplification at an expected band size of 670 bp as shown in Figure 1 and these were further used in the investigations. There was a positive amplification of the internally transcribed spacer region (ITS)1 and ITS4 regions which are known as conserved regions in fungi and are often used in their discrimination. The blasted sequences of fungal isolates showed high homology (90–100%) to known strains in the NCBI gene bank and helped to further identify the selected fungi strains as belonging to the *Aspergillus* genera. The percent diversity of selected fungi strains is as presented in Figure 2. The percentage occurrence of different species of *Aspergilli* ranged from 2.2–44.4%. *Aspergillus flavus* had the highest occurrence (44.4%) while *Aspergillus ochraceoroseus*, *Aspergillus tubingensis,* and *Aspergillus amstelodami* (2.2%) had the lowest. Other identified strains include; *Aspergillus oryzae* (8.8%), *Aspergillus niger* (6.67%), *Aspergillus terreus* (13.3%), *Aspergillus clavatus, Aspergillus tubingensis, Aspergillus nomius, Aspergillus fumigatus,* and *Aspergillus parasiticus* (4.44%).

### 2.2. Expression of Aflatoxin Biosynthesis Pathway Genes

**Figure 3:**
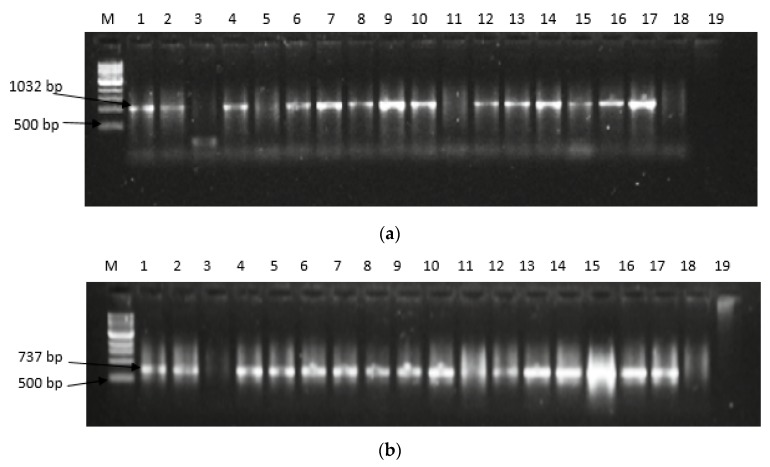

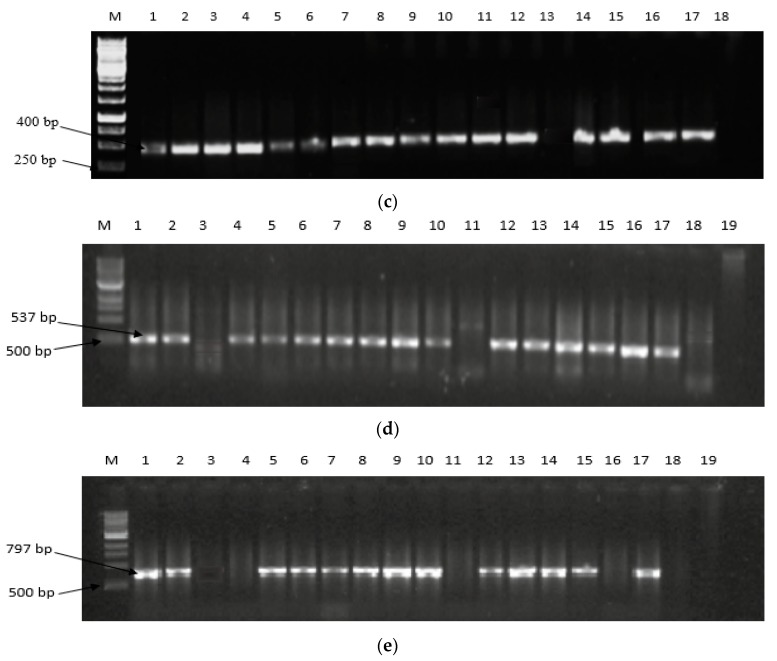
Gel electrophoresis patterns for expression of aflatoxin biosynthesis genes in representative *Aspergilli***.** (**a**) Gel electrophoresis patterns for expression of *afl*R genes at 1032 bp in *Aspergillus* strains isolated from feedlots of animals kept for food production. Lane M = DNA marker (1 kb); Lane 1 to 17 = positive amplification; Lane 3, 5, and 11 = Negative amplification; Lane 18 = *Saccharomyces cerevisiae* (Negative control); and Lane 19 = No template (Internal control), Lane 17 = *Aspergillus flavus ATCC* 259622^TM^ (Positive control). (**b**) Gel electrophoresis patterns for expression of *afl*J genes at 737 bp in *Aspergillus* strains isolated from feedlots of animals kept for food production. Lane M = DNA marker (1 kb); Lane 1 to 17 = positive amplification; Lane 3 and 11 = Negative amplification; Lane 18 = *Saccharomyces cerevisiae* (Negative control); and Lane 19 = No template (Internal control), Lane 17 = *Aspergillus flavus ATCC* 259622^TM^ (Positive control). (**c**) Gel electrophoresis patterns for expression of *afl*D (*Nor*-1) genes at 400 bp in *Aspergillus* strains isolated from feedlots of animals kept for food production. Lane M = DNA marker (1 kb); Lane 1 to 17 = positive amplification; Lane 13 and 18 = Negative amplification; Lane 13 = *Saccharomyces cerevisiae* (Negative control); and Lane 18 = No template (Internal control), Lane 17 = *Aspergillus flavus ATCC* 259622^TM^ (Positive control). (**d**) Gel electrophoresis patterns for expression of *afl*M genes at 537 bp in *Aspergillus* strains isolated from feedlots of animals kept for food production. Lane M = DNA marker (1 kb); Lane 1 to 17 = positive amplification; Lane 3, 11, 18, and 19 = Negative amplification; Lane 18 = *Saccharomyces cerevisiae* (Negative control); and Lane 19 = No template (Internal control), Lane 17 = *Aspergillus flavus ATCC* 259622^TM^ (Positive control). (**e**) Gel electrophoresis patterns for expression of *omt*-A genes at 797 bp in *Aspergillus* strains isolated from feedlots of animals kept for food production. Lane M = DNA marker (1 kb); Lane 1 to 17 = positive amplification; Lane 3, 4, 11 and 16 = Negative amplification; Lane 18 = *Saccharomyces cerevisiae* (Negative control); and Lane 19 = No template (Internal control), Lane 17 = *Aspergillus flavus ATCC* 259622^TM^ (Positive control).

**Figure 4:**
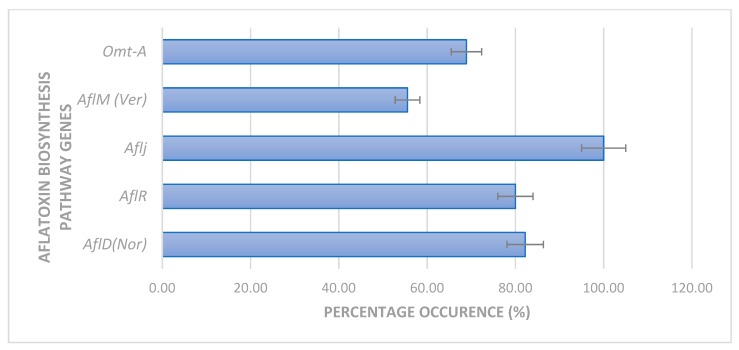
Percent amplification of aflatoxin biosynthesis pathway genes in selected *Aspergilli.*

Figure 3a–e presents the gel electrophoresis pattern of amplified aflatoxin regulatory genes in representative *Aspergilli*. Figure 3a presents a representative gel electrophoresis pattern of the amplified *afl*R gene. Successful amplification was obtained at an amplicon band size of 1032 bp in the representative *Aspergillus* strains and was comparable to the positive control (*Aspergillus flavus ATCC* 259622^TM^) while the negative control (*Saccharomyces cerevisiae*) had no amplification. However, some isolates had no amplification (FG1, FG7, FG8, FG16, FG29, and FG40). As shown in Figure 4, a total of 88.8% of the isolates had the *afl*R gene while no amplification was obtained in the negative internal control (DNA free water) and the other isolates. Figure 3b presents the gel electrophoresis patterns of *afl*J gene amplification in selected representative *Aspergilli*. Contrary to that in the *afl*R gene, 100% amplification was obtained in *afl*J in selected *Aspergillus* strains at 737 bp.

Furthermore, Figure 3c presents the gel electrophoresis patterns from the amplification of *afl*D (*Nor*-A) gene. A total of 86.4% had positive amplification (Figure 4) at an expected band size of 400 bp. The positive control was amplified while the negative controls was not. *Nor*-A gene was not detected in *Aspergillus sp*. (FG2), *Aspergillus terreus* (FG6 and FG11), *Aspergillus amstelodami* (FG13), and *Aspergillus tubingensis* (FG16) despite the expression of either *afl*R or *afl*J genes. Figure 3d presents the gel pattern for the amplification of *afl*M (*ver-1*) gene in representative *Aspergilli*. There was a total of 24 (58%) positive amplifications as shown in Figure 4. Amplification was observed at an expected band size of 735 bp except in the negative control and some *Aspergillus* strains; *Aspergillus flavus*, *Aspergillus niger*, *Aspergillus terreus*, *Aspergillus clavatus*, *Aspergillus amstelodami*, *Aspergillus fumigatus*, and *Aspergillus tubingensis*. Also, the gel electrophoresis pattern for the amplification of *omt*-A gene is as presented in Figure 3e. A total of 73% (Figure 4) of the *Aspergilli* had positive amplification at the expected amplicon size of 797 bp. However, no amplification was observed in 28% of the isolates and, as expected, in the negative control.

The detection of aflatoxins produced in selected fungal isolates was evaluated by the ammonium vapor test using the yeast extract sucrose medium. The production of yellow pigment around the colony, and detection of fluorescence under UV-light in the β-CDNRDCA media. As presented in Table 2, a total of 29 of the test isolates had a positive reaction to the ammonium vapor test, as shown by the production of a reddish brown ring around the fungal colony while 75.5% had yellow pigment production around the fungal colony in β-CDNRDCA. Twenty-six (57.7%) out of the total isolates had blue to blue-green fluorescence in β-CDNRDCA media when viewed under the UV-light at a wavelength of 365 nm. The degree of fluorescence in β-CDNRDCA ranged from no fluorescence (44.4%) to low (6.6%), mild (15.5%), high (20%), and very high fluorescence (4.44%). *Aspergillus nomius* (FG15) and *Aspergillus flavus* (FG14) were found to produce a very high fluorescence under the UV-light.

Furthermore, *Aspergillus clavatus* (FG27, FG35), *Aspergillus terreus* (FG37, FG38), and *Aspergillus flavus* (FG32 and FG45) had yellow pigment produced but failed to produce fluorescence in β-CDNRDCA under the UV-light despite the aflatoxin regulatory genes were positively expressed. *Aspergillus niger* (FG21) produced a yellow pigment and fluorescence while it produced no red pigment on ammonium vapor test. *Aspergillus flavus* (FG39) had a positive reaction in ammonium vapor test but failed in the production of yellow pigment on the yeast extract sucrose medium (YES) medium. *Aspergillus oryzae* (FG1) and *Aspergillus flavus* (FG2) had positive reaction in ammonium vapor test, produced a yellow pigment but failed to fluoresce on β-CDNRDCA.

**Figure 5:**
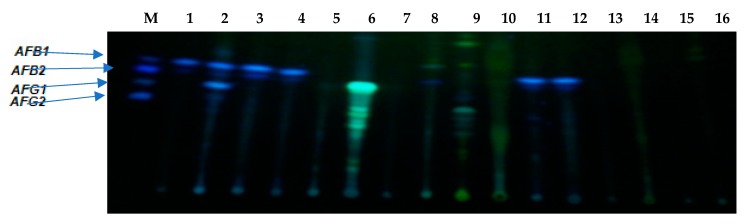
Detection of aflatoxin production in *Aspergilli* isolates from feedlots of animals kept for food production using the thin-layer chromatography. Lane M = Aflatoxin Standard (AFB1, AFB2, AFG1, and AFG2); Lanes 1–10 = Positive detection; Lanes 11 = Positive Control (toxigenic *Aspergillus flavus*); Lanes 13 = Negative detection; Lanes 14 = *Saccharomyces cerevisiae*; and Lanes 15 = Negative control (Nuclease free water).

The thin-layer chromatography (TLC) detection was conducted on all selected *Aspergillus* isolates. Nineteen (42.2%) out of the 45 selected strains tested positive for the production of aflatoxins. AFB1 and AFB2 production were shown by a blue fluorescence while AFG1 and AFG2 were confirmed by a blue-green fluorescence at a similar migration pattern to the standards. Figure 5 presents the results of aflatoxin detection in wheat flour inoculated with presumptive toxigenic species of *Aspergilli* using the TLC. The production of aflatoxins (AFB1, AFB2, AFG1, and AFG2) was detected in the *Aspergillus* species inoculated in wheat flour. Lanes 5 and 8 showed detection of another toxin aside those assayed (AFB1, AFB2, AFG2, or AFG1) in this study. No toxin was detected in the negative and the internal controls while there was the detection of AFB1 and AFB2 in the positive control (*Aspergillus flavus*) thus signifying a reliable result.

*Aspergillus flavus* (FG4) and *Aspergillus oryzae* (FG34 and FG36) isolates that have shown capabilities to produce aflatoxins through the ammonium vapor test and fluorescence on β-CDNRDCA were not detected on the TLC. Also, the non-detection of aflatoxin in *Aspergillus flavus* (FG9 and FG4) and *Aspergillus oryzae* (FG34 and FG36) on TLC and YES medium despite the potentials displayed in the molecular assay could be due to the low concentration of aflatoxin, below the optimum level of detection on the TLC plates.

A total of 23 isolates were selected for further aflatoxin quantification using the high-performance liquid chromatography (HPLC). Presumptive toxigenic *Aspergillus* strains were selected based on the revealed potentials for aflatoxin production through the molecular, conventional, and TLC assays. Table 3 presents the quantity of aflatoxins produced by selected *Aspergilli* inoculated in wheat flour substrate (µg/g). The concentration of AFG2 produced by selected *Aspergilli* ranged from 0.00 to 769 µg/g, AFG1 (0.00–745 µg/g), AFB2 (0.00–243 µg/g), AFB1 (0.00–7029 µg/g), and total aflatoxin (AFTOT) (0.00–71,454 µg/g). The AFG2 production was highest in *Aspergillus nomius* (FG15) while AFG1 was highest in *Aspergillus parasiticus* (FG12). Similarly, *Aspergillus parasiticus* (FG12) produced the highest AFB2 concentration (243 µg/g) while *Aspergillus nomius* (FG15) produced the highest AFB1 (70,289 µg/g) and AFTOT (71,454 µg/g). AFB2, AFG2, and a negligible amount of AFG1 and AFB1 was produced by *Aspergillus ochraceoroseus*. Furthermore, *Aspergillus oryzae* (FG25) produced both AFG2 and AFB2 only while *Aspergillus nomius* (FG43) and *Aspergillus ochraceoroseus* (FG44) produced all the aflatoxins assayed.

The standard calibration curves for AFB1, AFB2, AFG1, AFG2, and AFTOT gave an excellent linearity with the highest coefficient of regression (*R^2^*) of one. The limit of detection (LOD) was 10 µg/g, the limit of quantification (LOQ) was 20 µg/g while the per cent recovery calculated from the matrix-effect and slope was 85%. The assayed aflatoxins were eluted at the following retention time; AFG2 (5.5–6.5 min), AFG1 (6.5–8.0 min), AFB2 (8.0–9.5 min), and AFB1 (10.0–11.5 min). The AFG1 quantification on linearization followed the regression equation y = 3E + 06x while AFG2 (y = 9E + 06x), AFB2 (y = 4E + 06x), AFB1 (y = 7E + 06x), and AFTOT (y = 2E + 07x). The aflatoxin production by different *Aspergillus* strains was significant at a 95% confidence interval. AFG2 production among the *Aspergillus* spp. showed a significant difference (*p* ≤ 0.05) in the isolates compared to the controls. Also, a significant difference existed between the AFG1 concentration of FG12, FG15, FG10, FG31, FG 44, FG22, and FG43 isolates while no significant difference was obtained in the control and other isolates.

Furthermore, FG10 and FG4 were not significantly different from each other in terms of total aflatoxin production (AFTOT). Likewise, FG22 and FG7, FG24 and FG14, and FG20 and FG42 had no significant difference at *p* ≤ 0.05 but differs with reference to aflatoxin production in the controls and other *Aspergillus* species investigated. The isolates FG7, FG19, FG22, FG40, FG42, and positive control (*Aspergillus flavus*) had AFG2 concentration below the limit of detection while isolates FG10, FG22, FG31, FG43, and FG44 had AFG1 concentrations below the LOD. Similarly, isolates FG4 and FG44 had AFB2 and AFB1 concentrations below the LOD, respectively.

## 3. Discussion

Fungi belonging to the group *Aspergillus, Penicillium,* and *Fusarium* [29] have been implicated for mycotoxin production in food and feed. This could be as a result of their supposed dominance in agricultural commodities which serves as food for both humans and animals. It is noteworthy that prior to mycotoxin production in food/feed substrate, spore growth of fungal colonies having the capacity to produce mycotoxin is critical. Notably, some *Aspergillus* species that have been reported to have the ability to produce aflatoxin includes; *A. flavus*, *A. parasiticus*, *A. nomius*, *A. bombycis*, *A. ochraceoroseus*, *A. pseudotamarii*, *A. parvisclerotigenus*, *A. rambellii,* and *A. tamarii* [22]. However, some other groups of fungi have recently been implicated to produce aflatoxin of which many fungi of the genus *Aspergillus* are the most abundant in the tropical part of the world [30,31] and are readily involved in food spoilage. Furthermore, animal feeds, such as hay and straw, can be contaminated with pathogenic fungi which are abundant in the environment during pre- or post-harvest operations [1]. As obtained in this study, a large portion of *Aspergilli* obtained from feedlots of animals belongs to the species *A. flavus* (42.2%). This supports the report on *A. flavus* as the most common and prevalent *Aspergillus* species in nature, and has been described as the main fungal contaminants in African foods and feeds [30].

The amplification of the *afl*R and *afl*J genes in *Aspergillu*s species is indicative of the presence of aflatoxin regulatory genes. The *afl*R gene has been reported to mediate in the initiation of the transcript for aflatoxin biosynthesis pathway. However, the presence of *afl*R and *afl*J gene does not necessarily imply the capability of fungi to produce aflatoxin as the AF pathway is governed by many mechanisms [21]. Some previously known atoxigenic strains such as *A. oryzae* and *A. sojae* have been reported to possess the *afl*R gene [32] and similar morphological characteristics to *Aspergillus flavus* and *Aspergillus parasiticus* [21].

Furthermore, *afl*J is another regulatory gene in the aflatoxin biosynthesis pathway that is important for the expression of other aflatoxin biosynthesis genes which are responsible for the conversion of primary metabolites to aflatoxin [33]. However, despite the presence of *afl*J in the aflatoxin biosynthesis pathway, its role is still unclear. *afl*R and *afl*J are two genes with individual promoters that could be separately expressed in fungi. A similar observation was observed in this study in that some *Aspergillus* species were found to possess both the *afl*R and *afl*J aflatoxin regulatory genes. This observation corroborates the report on the expression of *afl*R and *afl*J in some *Aspergillus* isolates from Cashew nut [21]. The expression of *afl*R or *afl*J gene still calls for concern as these genes are indicative that such fungi could later acquire and express some other aflatoxin genes. Unfavorable conditions such as substrate, pH, temperature, water activity, plant metabolites, and light are factors that predisposes aflatoxin biosynthesis [34,35] likewise evolution and competition within the eco-system.

Also, the non-amplification of *Nor-*A gene in FG2, FG6, FG11, FG13, and FG16 may be due to the presence of a non-functional *afl*R or *afl*J genes, mutation as a result of insertion or deletion in the promoters needed for reads in *Nor*-A gene amplification. *Nor*-A gene is made up of a protein known as ketoreductase which converts AFB1 intermediate (Norsolorinic acid) into averantin. This protein is found in the cytosol of the fungal vegetative cell. The *afl*D gene is responsible for the expression of some other aflatoxin intermediates (averantin, verscolorin, and sterigmatocystin) along the AF’s pathway [36]. The none amplification of the *Ver-1* gene in some *Aspergillus* species could be due to a frame shift in the genome or gene mutation which could be a function of exposure to adverse environmental condition [32]. The *afl*M is a gene known for the conversion of averantin to versicolorin-A in the aflatoxin biosynthesis pathway [37]. However, some isolates portrayed the ability to produce other metabolites in the flour substrates hence, further investigation on the metabolomic profiles of the *Aspergilli* inoculated into flour substrates could be necessary. Several studies have reported the links between the sterigmatocystin, aflatoxin production, and fungal growth. The *omt*-A gene in *Aspergillus* strains is crucial to aflatoxin production especially in toxigenic *Aspergillus* species except in *Aspergillus nidulans* and *Aspergillus terreus* [34,38]. The *omt*-A gene is responsible for the conversion of sterigmatocystin to o-methylsterigmatocystin [21] which is critical to aflatoxin production. Furthermore, some isolates have been evaluated to possess all the aflatoxin regulatory genes and the necessary enzymatic processes leading to sterigmatocystin production but yet having no ability to produce o-methylsterigmatocystin [33]. A similar observation was obtained in this study (Table 2) where *Aspergillus flavus* (FG45 and FG39) and *Aspergillus oryzae* (FG1) possess *afl*R, *afl*J, *afl*D, and *afl*M but lack the *omt*-A gene leading to sterigmatocystin production. Therefore, this study affirms that the presence of o-methylsterigmatocystein does not necessarily confirms aflatoxin production.

Notably, in *Aspergillus terreus* (FG17) *afl*M (*ver-1*) and *omt*-A gene were not detected, this could be due to inadequate environmental conditions which could have caused a mutation of the *omt*-A and *afl*M gene in the fungal pathogen. This observation might be due to a distortion in the order of enzyme production leading to aflatoxin biosynthesis. The several transcription factors such as pH, carbon, or nitrogen source hold the ability to affects chromatin organization by either inhibiting or activating gene expression. These factors could either support the binding or mismatching of sequence-specific primers to sites in the promoter regions of the target genes thereby forming complexes that aid another form of transcription process [38]. Another factor that could affect the expression of aflatoxin biosynthesis genes is the location of the chromosome in the genome of presumptive toxigenic fungi [39]. The presence of hexose utilization gene cluster next to the aflatoxin biosynthesis pathway could interfere with the aflatoxin production pathway since aflatoxin can be induced by simple sugars such as glucose and sucrose [40].

As observed in this study, the conventional method (YES and β-CDNRDCA) aided the effective discrimination of the toxigenic strains of *Aspergilli* compared to the sole use of the molecular assay. However, this observation is in agreement with the previous reports on the inadequacy of the use of only molecular method in the identification of aflatoxin producers [21,29,41,42]. The formation of only yellow pigment and fluorescence by *Aspergillus* strains have been reported not to be a reliable means of identifying aflatoxin-producing strains of *Aspergilli*. Hence, the need to combine both the conventional with the molecular method. The observation of the failure of isolates FG27, FG35, FG37, FG38, FG32, and FG45 to produce fluorescence under UV-light despite the positive expression of aflatoxin biosynthesis pathway genes is in conformity with previous reports [21,42] where authors reported a similar observation in some *Aspergillus* strains isolated from Cashew nuts, millet, and sesame grains in Nigeria, respectively. A bright blue fluorescence is usually achieved when a reaction exists between a produced aflatoxin and hydrophobic β-cyclodextrin which is capable of fluorescing under UV-light [43]. The non-fluorescence of fungal isolates in β-CDNRDCA might be due to low concentration of aflatoxin produced in the test medium making its capture under the UV-light insignificant. Some atoxigenic strains of *Aspergilli* have been reported to show the ability to produce yellow pigment while not possessing the ability to produce aflatoxins [44]. The yellow pigment and ammonium hydroxide vapor observed on the reverse side of the β-CDNRDCA and YES plates respectively is an anthraquinone biosynthetic intermediates (Averufin) which are produced along the aflatoxin biosynthesis pathway often found between norsolorinic acid (*Nor*) and versicolorin A [45].

Also, the non-detection of aflatoxin in *Aspergillus flavus* (FG9 and FG4) and *Aspergillus oryzae* (FG34 and FG36) on TLC and YES medium could be due to unsuitable substrates that could aid the production of the secondary metabolites. Source of nutrient (carbon or nitrogen) could affect the growth rate of fungal species which could alter the aflatoxin production kinetics in toxigenic strains [39]. *Aspergillus flavus* is known to be a chief producer of AFB1 and/or AFB2 toxins, however, in this study *Aspergillus flavus* (FG9) was found not to produce either of the two toxins in wheat flour substrate. This could either be due to the low level of aflatoxin production below the level of detection or lack of favorable conditions such as a substrate for growth, moisture content, temperature, and pH to aid growth and production of aflatoxins. The variation in the aflatoxin-producing ability of *Aspergillus flavus* (FG9) supports the findings of Abbas, Zablotowicz [44] on *Aspergillus flavus*.

Furthermore, *Aspergillus oryzae* (FG34 and FG36) did not produce aflatoxin as shown in Table 3 despite its ability shown in the conventional assays. This finding supports the previous report that *Aspergillus oryzae* are a domesticated type of *Aspergillus flavus* and are known to be atoxigenic. There exists a positive correlation between the TLC and HPLC methods used in the detection of aflatoxin in selected fungi. In recent times, the occurrence of aflatoxin producers among the *Aspergillus* genera has been on the increased giving rooms to some strains not previously characterized as aflatoxin producers. *Aspergillus nomius*, *Aspergillus bombycis*, *Aspergillus ochraceoroseus,* and *Aspergillus pseudotamarii* has been described to produce aflatoxin occasionally [22], thus supporting the findings in this study. Aflatoxin production is an undesirable activity of the *Aspergillus* genera that poses a huge threat to the safety and health of humans and veterinary. In this study, it was observed that some *Aspergillus flavus* isolates (FG18, FG19, FG20, FG24, and FG33) hold the ability to produce AFB and trace amount of AFG’s, against the previous report of its production exclusively by *Aspergillus parasiticus*. The production of the aflatoxin G’s by some strains previously known as non-aflatoxin producers could be due to genetic interaction between the toxigenic and atoxigenic strains of fungi within the eco-system. A high gene similarity has been reported along the sterigmatocystin pathway of *Aspergillus flavus* and *Aspergillus parasiticus* [40]. Similarly, *Aspergillus oryzae* and *Aspergillus sojae* have been shown to be closely related to *Aspergillus flavus* and *Aspergillus parasiticus* but yet to be reported as aflatoxin producers [46]. The inability of *Aspergillus oryzae* and *Aspergillus sojae* to produce aflatoxin has been explained to be due to gene deletion or mutations that resulted in the silencing of the aflatoxin biosynthesis gene [47]. However, *Aspergillus oryzae* and *Aspergillus sojae* holds the potentials to produce aflatoxin. This assertion was based on the fact that they possess a similar aflatoxin biosynthesis pathway genes as observed in *Aspergillus flavus* and *Aspergillus parasiticus*. We suggest the production of aflatoxin by *Aspergillus oryzae* in this study might be due to a lateral gene transfer between the genome of aflatoxin-producing strains and previously known atoxigenic strains colonizing the feedlots of animals that are kept for food. Therefore further studies could be conducted to investigate gene alteration and mutations in fungal isolates as a result of extreme environmental conditions or as a product of microbial interactions.

The closer the R^2^ is to one the higher the accuracy of a determination. Hence, the high R^2^ value obtained in this study depicts the accuracy of the aflatoxin determinations. The concentrations of aflatoxins produced by some of the fungal pathogens were below the LOD which is desired, however, the concentrations might increase if pathogens are exposed to more favorable growth conditions. The total aflatoxin production reported in this study are above the European Union tolerable limits for aflatoxin (4 µg/kg) and the legislated aflatoxin limits of 20 μg/kg in animal feeds. The Fertilizers Farm, Feed, Agricultural Remedies and Stock Remedies Acts (Act No. 36 of 1947) stipulates 10 µg/kg standards for aflatoxins (South African Government 2009) while standards of 20 ppb set by some countries were exceeded in this present study. The Federal Drug Agency USA have set a tolerable limit of aflatoxin in feedstuff at 1000 µg/kg (Kubo, 2012). Toxigenic strains of fungi belonging to the Flavi family are known as producers of aflatoxins which could have a negative effect on the health of both animals and human [48] when ingested in food. *Aspergillus flavus* and *Aspergillus parasiticus* have been grouped as a group one carcinogen by the International Agency for Research on Cancer [49] and rated as the major cause of liver cancer. The incidence of aflatoxin in animal feeds has been reported in South Africa, Kenyan, Sudan, Morocco, and Nigeria [50,51,52,53,54,55].

## 4. Conclusions

This study supports the use of a polyphasic approach in the evaluation of toxin producing potentials of fungal species as a single approach might not give a reliable insight into the toxin production ability of presumptive toxigenic fungi. The fluorescence assays and chromatography approach could serve as a confirmatory assay to the molecular detection assays. In this study, some *Aspergillus flavus* isolates from feedlots of animals were found to be non-aflatoxigenic. *Aspergillus oryzae*, *Aspergillus ochraceoroseus,* and *Aspergillus nomius* could produces both the B and G aflatoxins. This study reports for the first time the potentials of *Aspergillus oryzae* to produce aflatoxin, however, at a trace level. *Aspergillus* species colonizing the feedlots of animals kept for food production in Mafikeng, North West Province, South Africa are capable of producing aflatoxin at a concentration higher than the stipulated European Union legislated standards in food and feeds. The finding from this study show a possibility for aflatoxicosis conditions in the North West Province as this could impact negatively on the socioeconomic life, food security, safety, and health of animals and humans. Therefore, there should be increased surveillance for aflatoxin contaminations in agricultural commodities used for food and livestock rearing in the North West Province, South Africa.

## 5. Materials and Methods

### 5.1. Materials

Filamentous fungi belonging to genus *Aspergillus* that were used in this study were isolated from feeds of animals reared for food production and were collected from the Toxicological/Biochemistry Laboratory in the Department of Animal Health, North-West University, Mafikeng Campus, South Africa. Whole wheat flour was purchased from some randomly selected supermarkets in Mafikeng, North West Province, South Africa. *Aspergillus flavus* and *Saccharomyces cerevisiae* were used as positive and negative control strains, respectively. Purified aflatoxin B1, aflatoxin B2, aflatoxin G1, and aflatoxin G2 were used as standards in this study and were procured from Sigma Aldrich, St Loius, MO, USA. All chemical reagents used in this study were of analytical grade and were procured from both Sigma Aldrich, St Loius, MO, USA and Merck Chemicals Pty Ltd., Wadeville, Gauteng, South Africa and Biolab, Modderfontein, South Africa.

### 5.2. Culturing of Fungi

Forty-five (45) fungi isolates were selected out of the lots from the fungal collection in the Toxicological/Biochemistry Laboratory of the Department of Animal Health, North-West University, Mafikeng Campus, South Africa. Criteria for selection was based on morphological characteristics similar to *Aspergillus* species. Therefore, isolates presumably belonging to *Aspergillus* genus were re-activated in malt extract broth and were cultured aseptically. The colonies were purified on potato dextrose agar. Isolates showing green, blue-green, green and black pigmentation on Potato Dextrose Agar were selected and cultured using a surface point inoculation on sterile PDA plates and incubated for 72 h at 25 ± 2 °C.

### 5.3. Molecular Identification of Presumptive Aspergilli

Mycelia were harvested from fungal isolates and used for DNA extraction using the DNA^TM^ Fungal/Bacterial Miniprep extraction kit (Zymo Research Corporation, Irvine, CA, USA) following the manufacturer’s instructions. The pure genomic DNA of fungi isolates were quantified using a Nanodrop Lite spectrophotometer (Model 1558) obtained from Thermo Scientific, Wilmington, DE, USA. The concentration (ng/µL) of extracted DNA was measured using the absorbance at 260 nm and the purity was determined at 260/280 nm. Isolates with absorbance ratio ≥1.8 were considered pure. The presence of DNA was confirmed through a 1% (*w/v*) agarose gel electrophoresis and analysis was conducted at 400 A, 80 V for 30 min. The gel was then viewed under the UV Transilluminator (Biorad Gel Doc^TM^ XR+ Philadelphia, PA, USA) to confirm the presence of DNA. The eluted DNA was stored at −80 °C for further molecular identification assays.

#### PCR Amplification of ITS1 and ITS4 of Presumptive Aspergillus Isolates

The amplification of internally transcribed spacer regions, ITS1 and ITS4, that facilitate identification of *Aspergillus* isolates was performed using previous protocols [56]. The amplification was conducted in a Biorad C1000 Touch^TM^ Thermal Cycler. The *ITS*1–*ITS*4 forward (5′-TCC GTA GGT GAA CCT GCG G-3′) and reverse (5′-TCC TCC GCT TAT TGA TAT G-3′) oligonucleotide primers was synthesized at Inqaba Biotechnical Industries (Pty) Ltd., Pretoria, South Africa. The PCR was performed as standard 25 µL volumes comprising 1X PCR master mix, 50 *pmol* of primers, 4 µL of template DNA, and nuclease free water. The PCR mix consumables were obtained from ThermoFischer Scientific, Wilmington, DE, USA. DNA extracted from an environmental *Saccharomyces cervisiae* and *Aspergillus flavus* strains was used as negative and positive controls respectively during amplification reactions. PCR amplification conditions comprised an initial denaturation of 95 °C for 5 min, 35 cycles of denaturation at 94 °C for 30 s, annealing at 61 °C for 30 s, elongation at 72 °C for 5 min, and a final elongation at 72 °C for 7 min. PCR amplicons were held at 4 °C until electrophoresis. PCR amplicons were resolved on a 1% (w/v) agarose gel containing 0.25 μg/mL ethidium bromide. Electrophoresis was conducted at 60 volts, 400 amperes (A) for 60 min using an electrophoresis units (Bio-Rad Laboratories, CA, USA) containing 1% (v/v) TAE buffer (Fermentas Life Science, Vilnius, Lithuania). Each gel contained a 1 kb DNA molecular weight marker (Fermentas Life Science, Vilnius, Lithuania) that was used to confirm the size of PCR fragments. Gels were visualized under the UV transilluminator gel documentation unit (Biorad Gel Doc^TM^ XR+, Philadelphia, PA, USA) and images were recorded. Amplified ITS gene fragments were sequenced by Inqaba Biotec Ltd., Pretoria, South Africa. Blast searches (http://www.ncbi.nlm.nih.gov/BLAST) were used to confirm the identities of the sequences and accession numbers were obtained.

### 5.4. Expression of Aflatoxin Biosynthesis Pathway Genes in Filamentous Aspergilli

The expression of the aflatoxin biosynthesis regulatory genes *afl*R, *afl*J, *aflD* (*Nor*-A), *aflM* (*ver*-1), and *omt*-A were evaluated as previously described [21]. Oligonucleotide primer sequences and amplifications conditions used are shown in Supplementary Table S1. Amplifications were performed in 25 µL reaction volumes comprising of 1X PCR master mix, 50 pM of forward and reverse primers, 4 µL of template DNA and nuclease free water using a Biorad C1000 Touch^TM^ Thermal Cycler. The PCR products were separated on a 1% (*w/v*) agarose gel containing 0.25 μg/mL ethidium bromide. The percent expression of aflatoxigenic genes was calculated as described in Equation (1). The toxigenic *Aspergillus* strains were then screened for aflatoxin production using an in vitro conventional method.

(1) $$\mathrm{Aflatoxigenic}\;\mathrm{genes}\;\left(\%\right)=\frac{\mathrm{No}\;\mathrm{of}\;\mathrm{positive}\;\mathrm{amplification}}{\mathrm{Total}\;\mathrm{number}\;\mathrm{of}\;\mathrm{selected}\;\mathrm{fungi}\;\mathrm{with}\;\mathrm{potential}\;\mathrm{for}\;\mathrm{mycotoxin}\;\mathrm{production}}\times 100$$

### 5.5. Evaluation of Filamentous Aspergillus spp. for Aflatoxin Production

#### 5.5.1. β-Cyclodextrin Neutral Red Desiccated Coconut Agar (β-CNRDCA) Assay

Spores of *Aspergillus* strains were grown overnight in malt extract medium at 28 ± 2 °C. A surface point inoculation was performed with a sterilized needle on β-cyclodextrin neutral red desiccated coconut agar (β-CNRDCA) [41]. Briefly, about 200 g of desiccated coconut was soaked in a liter of boiled purified distil water (pH 4.8) and was homogenized for 5 min in a high-speed blender. The mixture obtained was sieved and the filtrate was used in the preparation of the β-CNRDCA. An amount of 0.2% (*v/v*) of neutral red dye, 0.3% (*w/v*) β-cyclodextrin, and 2% of microbiological agar medium (Merck, Darmstadt, KGaA, Germany) was added to the filtrate to give a light pink color for improve fluorescence by aflatoxigenic strains. The resulting media was stirred and boiled on a gas burner and allowed to cool prior to sterilization in an autoclave at 121 °C for 15 min. The media was poured on 6 cm petri dish plates, allowed to solidify and plates were inoculation with selected fungi strains. Inoculated plates were incubated at 28 ± 2 °C for 14 days. Mycotoxin production was evaluated by the presence of yellow pigment and the detection of blue or bluish green fluorescence under ultraviolet light at 365 nm. *Aspergillus flavus* and *Saccharomyces cerevisiae* were used as positive and negative controls, respectively, while a β-CNRDCA plated without fungi inoculation served as negative internal control. The percent detection of mycotoxin on β-CNRDCA (β-CNRDCA %) was expressed as shown in Equation (2).

(2) $$\beta -\mathrm{CNRDCA}\;\left(\%\right)=\frac{\mathrm{No}\;\mathrm{of}\;\mathrm{positives}\;\mathrm{isolates}\;\mathrm{on}\;\beta -\mathrm{NRDCA}\;}{\mathrm{Total}\;\mathrm{number}\;\mathrm{of}\;\mathrm{selected}\;\mathrm{fungi}\;\mathrm{with}\;\mathrm{potential}\;\mathrm{for}\;\mathrm{mycotoxin}\;\mathrm{production}}\;\;\times 100$$

#### 5.5.2. Yeast Extract Sucrose (Ammonium Vapor) Test Assay

Yeast extract sucrose agar was used to evaluate the potential of selected *Aspergillus* species to produce aflatoxin [24]. The YES medium was compounded by measuring 150 g/L of Sucrose and 15 g/L of bacteriological agar to 20 g/L of Yeast Extract broth medium into a conical flask and the media was dissolved in a liter of purified water. The resulting mixture was sterilized at 121 °C for 15 min. The YES medium plates were then surface centered inoculated with respective fungi isolates and incubated for seven days at 25 ± 2 °C. The aflatoxin production was evaluated after period of incubation by examining for yellow pigment around the fungi and the production of red pigment after exposing plates to five ml ammonia (NH_3_) solvent. *Aspergillus flavus* and *Saccharomyces cerevisiae* were used as positive and negative controls respectively while a YES medium plate without fungi inoculation served as negative internal control. The percent detection of mycotoxin on YES medium (YES%) was expressed as shown in Equation (3).

(3) $$\mathrm{YES}\;\left(\%\right)=\frac{\mathrm{No}\;\mathrm{of}\;\mathrm{positives}\;\mathrm{on}\;\mathrm{YES}\;\mathrm{media}}{\mathrm{Total}\;\mathrm{number}\;\mathrm{of}\;\mathrm{selected}\;\mathrm{fungi}\;\mathrm{with}\;\mathrm{potential}\;\mathrm{for}\;\mathrm{mycotoxin}\;\mathrm{production}}\;\times 100$$

### 5.6. Aflatoxin Production by Aspergilli in Wheat Flour Using Thin-Layer Chromatography (TLC) Assay

Whole wheat flour was sterilized at 121 °C for 15 min and dried to an equilibrium moisture content of 10% in hot air oven at 65 °C. The flour moisture content was reduced to remove the free water that could aid microbial growth. The moisture content of dried wheat flour was determined using a previously described technique [57]. Briefly, 10 g of the dried wheat flour was weighed to represent W_1_ and placed into a pre-weighed empty Petri dish (W_2_) that was also placed in a preset oven at 105 °C and allowed to stand for 3 h. Dried samples were placed in a desiccator for cooling and these represented W_3._ This process was repeated until a constant weight of 10% moisture content was obtained. The moisture content was calculated as described in Equation (4). A portion of 50 g of the wheat flour was measured into a conical flask and 10% moisture of sterile DNA free water was added. One milligram of *Aspergillus* spores per 50 g of flour was weighed on a digital weighing balance and was inoculated into moistened flour. The moistened flour was stirred using a sterile glass rod and was cotton plugged after which incubation was done at a relative humidity of 50% for 15 days at 25 ± 2 °C.

(4) $$\%\;\mathrm{Moisture}\;\mathrm{Content}=\frac{W2-W3}{W1}\;\times \;100$$

A solid –liquid extraction method was employed for mycotoxin extraction from wheat flour [58] with slight modifications. Twenty-five grams (25 g) of inoculated flour was weighed into a glass beaker and 50 mL of methanol: Water (*w/v*; 18:2) solution and 1 g sodium chloride (NaCl) was added. The mycotoxin extraction was done in a dark room to prevent fluorescence under white light. The mixture was blended in a high speed rotary stirrer IKA at maximum speed to aid the release of mycotoxin into the solvent. The slurry was filtered using a Whattman No 1 filter paper (Maidstone, UK) and the filtrate was evaporated to dryness under dark environment while the residue was discarded. The dried filtrate was then reconstituted with 1000 µL of HPLC grade methanol (Sigma Aldrich, St Loius, MO, USA) and was filtered through a 0.22 µm MS Nylon Syringe Millipore filter (SIMPLEPURE, NY, USA) fitted to a sterile syringe. The volume of elute was adjusted to 4 mL with methanol in LC/LCMS vials (Shimadzu, GbHg, Germany).

The aflatoxin detection by the TLC method was performed using the protocol [44] with slight modifications. A glass baked silica gel coated (CaSO_4_) plates with 20 by 20 cm dimension of 250 μm thickness supplied by Merck (KGaA, Darmstadt, Germany) was used in the qualitative analysis of the samples. A 40 µL aliquot of the extract was spotted on the TLC plates against 0.5 µg/mL aflatoxin standards (AFB1, AFB2, AFG1, and AFG2). The mobile phase (TLC solvent) comprised chloroform: Ethyl acetate: Propan-2-ol at proportions of 90:5:5 (*v/v/v*). The spotted plates were placed in the TLC tank containing the mobile phase to allow for migration by a capillary action through the stationary phase (TLC silica). The separated TLC plates were viewed under UV-light at a wavelength of 365 nm and images were photographed and recorded. AFB1, AFB2 and AFG1, AFG2 migration along with the aflatoxin standards was identified respectively by a blue and blue green fluorescence on the plate. The percent detection of each aflatoxin was calculated using the formula described in Equation (5).

(5) $$\mathrm{TLC}\;\left(\%\right)=\frac{\mathrm{No}\;\mathrm{of}\;\mathrm{positives}\;\mathrm{on}\;\mathrm{TLC}}{\mathrm{Total}\;\mathrm{number}\;\mathrm{of}\;\mathrm{selected}\;\mathrm{fungi}\;\mathrm{with}\;\mathrm{potential}\;\mathrm{for}\;\mathrm{mycotoxin}\;\mathrm{production}}\;\;\times 100$$

### 5.7. Quantification of Aflatoxin by High-Performance Liquid Chromatography

The concentration of aflatoxins produced by selected *Aspergillus* species was quantified in a High-Performance Liquid Chromatography Shimadzu liquid chromatograph (Kyoto, Japan) equipped with a Jasco FP-920 fluorescence detector of 362 nm excitation wavelength and 425 nm emission wavelength respectively for AFB1 and AFB2 while AFG1 and AFG2 were quantified at 455 nm. The column used was a Hichrom column (4.6 mm by 150 mm) of 5 μm, while the derivatization reactor used was KOBRA Cell at a regimen of 100 μA. The guard cartridge and analytical cartridge used was Inertsil ODS-3 and ODS-3V, respectively. The injector was an autosampler with reodyne valve. In order to perform HPLC analysis, 100 μL of the sample extract was injected into the equipment while 0.00025, 0.0025, 0.25, 2.5, and 25 μg/mL of aflatoxin standards were used in validating the analysis. The separations of the chromatography peaks was done using a Hichrom column to which a pre-column of similar stationary phase has been fitted. The mobile phase was made up of water: Acetonitrile: Methanol; (3:1:1, *v/v/v*) pumped at 1.0 mL/min at an injection volume of 100 μL following an isocratic line-up. Aflatoxins standards (AFB1, AFB2, AFG1, AFG2, and AFTOT) purchased from Sigma Aldrich (Sigma, St. Louis, MO, USA) were reconstituted with sterile biopure water and concentrations of 2 μg/mL each for AFB1 and AFG1 and 0.5 μg/mL each for AFB2 and AFG2 were prepared. Aflatoxin detection was regarded positive for each peak at a retention time similar to each standard and at a height five times higher than the baseline noise [25,54,58].

The HPLC method was validated by determining the accuracy, linearity, and sensitivity of the standards. The linearity was obtained by constructing a calibration curves from the emissions obtained from AFB1, AFB2, AFG2, AFG1, AFTOT, and the control which is made up of acetonitrile (HPLC grade). The calibration curves were plotted using five different concentrations of standards ranging from 0.0025 μg/mL to 25 μg/mL against the areas of the peaks (aflatoxin concentrations) while a regression analysis was carried out to determine linearity of the determination. The slope of the standard calibration curve and matrix-matched calibration curve for each concentration of the standards was used to determine the matrix effect (ME) of each concentration of analyte (aflatoxin). The matrix effect calibration curves was created by spiking blank sample (Acetonitrile) with aflatoxin standards.

On the other hand, the sensitivity of the HPLC system was determined by the limit of quantification and limit of detection. The LOQ was calculated by multiplying the signal to noise ratio by three while the LOD was calculated by the product of ten and the concentration obtained at the lowest level of chromatographic peaks of the spiked test samples. The recovery analysis was used to evaluate the accuracy of the quantification of aflatoxin produced by the fungal isolates. The recovery was obtained by determining the ratio of the area under chromatographic peaks of individual spiked aflatoxin standards obtained before extraction and after extraction. The recovery was done at three levels (25, 50, and 100 μg/kg) in addition to the previous standard concentration spiked.

### 5.8. Statistical Analysis

The statistical package for social sciences (SPSS, Inc., Chicago, IL, USA) version 21 was used in the analysis of aflatoxin produced by selected fungal isolates through the One-way analysis of variance (ANOVA). The means were separated using the Duncan new multiple range test at 95% confidence interval while the measure of central tendency (mean) and descriptive statistics (percentages) was used to express the aflatoxin production potentials of *Aspergillus* species isolated from feedlots of animals kept for food production.

## Figures and Tables

**Table 1:** Morphology of the fungi cultures isolated from feedlots of animals kept for food production.

SN	ID	Isolates Name	Assession No	Media	Colour and Morphology
1	FG1	*Aspergillus oryzae*	MG647833	MEA	Line Green
2	FG2	*Aspergillus flavus*	MG659619	MEA	Line green
3	FG3	*Aspergillus niger*	MG647838	MEA	Black
4	FG4	*Aspergillus flavus*	MG659628	MEA	Green
5	FG5	*Aspergillus niger*	MG647849	MEA	Black
6	FG6	*Aspergillus terreus*	MG647840	MEA	Dark green
7	FG7	*Aspergillus flavus*	MG659631	MEA	Line green
8	FG8	*Aspergillus tubingensis*	MG647844	MEA	Green
9	FG9	*Aspergillus flavus*	MH270531	MEA	Green
10	FG10	*Aspergillus flavus*	MG647845	MEA	Green
11	FG11	*Aspergillus terreus*	MG647846	MEA	Dark green
12	FG12	*Aspergillus parasiticus*	MG659626	MEA	Green
13	FG13	*Aspergillus amstelodami*	MG647851	MEA	Line Green
14	FG14	*Aspergillus flavus*	MH270612	MEA	Green
15	FG15	*Aspergillus nomius*	MG659621	MEA	Green
16	FG16	*Aspergillus tubingensis*	MG647853	MEA	Military green
17	FG17	*Aspergillus terreus*	MG647863	MEA	dark green
18	FG18	*Aspergillus flavus*	MG659635	MEA	Green
19	FG19	*Aspergillus flavus*	MH270544	MEA	Green
20	FG20	*Aspergillus flavus*	MG659673	MEA	Green
21	FG21	*Aspergillus niger*	MG647867	MEA	Black
22	FG22	*Aspergillus flavus*	MH270559	MEA	Green
23	FG23	*Aspergillus flavus*	MG659626	MEA	Green
24	FG24	*Aspergillus flavus*	MH270559	MEA	Green
25	FG25	*Aspergillus oryzae*	MH270563	MEA	Green
26	FG26	*Aspergillus flavus*	MH270574	MEA	Green
27	FG27	*Aspergillus clavatus*	MG647850	MEA	Line Green
28	FG28	*Aspergillus fumigatus*	MG647855	MEA	Bluish green
29	FG29	*Aspergillus fumigatus*	MG647869	MEA	Bluish green
30	FG30	*Aspergillus terreus*	MG647840	MEA	Dark green
31	FG31	*Aspergillus flavus*	MH270578	MEA	Green
32	FG32	*Aspergillus flavus*	MG647868	MEA	Green
33	FG33	*Aspergillus flavus*	MH270581	MEA	Green
34	FG34	*Aspergillus oryzae*	MG659690	MEA	Military green
35	FG35	*Aspergillus clavatus*	MG647856	MEA	Green
36	FG36	*Aspergillus oryzae*	MG659633	MEA	Green
37	FG37	*Aspergillus terreus*	MG647866	MEA	Green
38	FG38	*Aspergillus terreus*	MG647852	MEA	Green
39	FG39	*Aspergillus flavus*	MG647857	MEA	Green
40	FG40	*Aspergillus flavus*	MG659627	MEA	Green
41	FG41	*Aspergillus parasiticus*	MG659687	MEA	Green
42	FG42	*Aspergillus flavus*	MG659676	MEA	Green
43	FG43	*Aspergillus nomius*	MH270600	MEA	Green
44	FG44	*Aspergillus ochraceoroseus*	MH270530	MEA	Green
45	FG45	*Aspergillus flavus*	MG647871	MEA	Green
46	Control 1	*Aspergillus flavus*	ATCC 259622^TM^	MEA	Green
47	Control 2	*Saccharomyces cerevisiae*		MEA	Creamy

Keys: FG1–FG45 = fungal isolates, MEA = Malt extract agar.

**Table 2:** Polyphasic characterization for aflatoxin production in selected fungi isolates.

SN	ID	Isolates Name	Assession No	*afl*D (*Nor*-A)	*afl*R	*afl*J	*afl*M (*ver*-1)	*Omt*-A	YES (NH4 Vapor Test)	(β-CDNRDCA)		TLC	HPLC	Selected Isolates
										Yellow Pigment	UV-Florescence			
1	FG1	*Aspergillus oryzae*	MG647833	POS	NEG	POS	POS	NEG	POS	POS	-	nd	nd	
2	FG2	*Aspergillus flavus*	MG659619	NEG	POS	POS	NEG	POS	POS	POS	-	nd	nd	
3	FG3	*Aspergillus niger*	MG647838	POS	POS	POS	NEG	POS	NEG	NEG	-	nd	nd	
4	FG4	*Aspergillus flavus*	MG659628	POS	POS	POS	POS	POS	POS	POS	±	NEG	POS	**
5	FG5	*Aspergillus niger*	MG647849	POS	POS	POS	NEG	POS	NEG	NEG	-	nd	nd	
6	FG6	*Aspergillus terreus*	MG647840	NEG	POS	POS	NEG	POS	NEG	NEG	-	nd	nd	
7	FG7	*Aspergillus flavus*	MG659631	POS	NEG	POS	POS	POS	POS	POS	+++	POS	POS	**
8	FG8	*Aspergillus tubingensis*	MG647844	POS	NEG	POS	POS	NEG	NEG	NEG	-	nd	nd	
9	FG9	*Aspergillus flavus*	MH270531	POS	POS	POS	POS	POS	NEG	NEG	-	NEG	POS	**
10	FG10	*Aspergillus flavus*	MG647845	POS	POS	POS	POS	POS	POS	POS	+	NEG	POS	**
11	FG11	*Aspergillus terreus*	MG647846	NEG	POS	POS	NEG	NEG	NEG	POS	++	nd	nd	
12	FG12	*Aspergillus parasiticus*	MG659626	POS	POS	POS	POS	POS	POS	POS	+++	POS	POS	**
13	FG13	*Aspergillus amstelodami*	MG647851	NEG	POS	POS	NEG	NEG	NEG	NEG	-	nd	nd	
14	FG14	*Aspergillus flavus*	MH270612	POS	POS	POS	POS	POS	POS	POS	++++	POS	POS	**
15	FG15	*Aspergillus nomius*	MG659621	POS	POS	POS	POS	POS	POS	POS	++++	POS	POS	**
16	FG16	*Aspergillus tubingensis*	MG647853	NEG	NEG	POS	NEG	POS	NEG	NEG	-	nd	nd	
17	FG17	*Aspergillus terreus*	MG647863	POS	POS	POS	NEG	NEG	NEG	NEG	-	nd	nd	
18	FG18	*Aspergillus flavus*	MG659635	POS	POS	POS	POS	POS	POS	POS	+++	POS	POS	**
19	FG19	*Aspergillus flavus*	MH270544	POS	POS	POS	POS	POS	POS	POS	+++	POS	POS	**
20	FG20	*Aspergillus flavus*	MG659673	POS	POS	POS	POS	POS	POS	POS	+++	POS	POS	**
21	FG21	*Aspergillus niger*	MG647867	POS	POS	POS	NEG	NEG	NEG	POS	++	nd	nd	
22	FG22	*Aspergillus flavus*	MH270559	POS	POS	POS	POS	POS	POS	POS	++	POS	POS	**
23	FG23	*Aspergillus flavus*	MG659626	POS	POS	POS	POS	POS	POS	POS	+++	POS	POS	**
24	FG24	*Aspergillus flavus*	MH270559	POS	POS	POS	POS	POS	POS	POS	+/-	POS	POS	**
25	FG25	*Aspergillus oryzae*	MH270563	POS	POS	POS	POS	POS	POS	POS	+/-	POS	POS	**
26	FG26	*Aspergillus flavus*	MH270574	NEG	POS	POS	POS	NEG	POS	NEG	-	nd	nd	
27	FG27	*Aspergillus clavatus*	MG647850	POS	POS	POS	NEG	NEG	NEG	POS	-	nd	nd	
28	FG28	*Aspergillus fumigatus*	MG647855	NEG	POS	POS	NEG	POS	NEG	NEG	-	nd	nd	
29	FG29	*Aspergillus fumigatus*	MG647869	POS	POS	POS	NEG	POS	NEG	POS	-	nd	nd	
30	FG30	*Aspergillus terreus*	MG647840	POS	POS	POS	NEG	NEG	NEG	NEG	-	nd	nd	
31	FG31	*Aspergillus flavus*	MH270578	POS	POS	POS	POS	POS	POS	POS	++	POS	POS	**
32	FG32	*Aspergillus flavus*	MG647868	POS	POS	POS	NEG	NEG	NEG	POS	-	nd	nd	
33	FG33	*Aspergillus flavus*	MH270581	POS	POS	POS	POS	POS	POS	POS	++	POS	POS	**
34	FG34	*Aspergillus oryzae*	MG659690	POS	POS	POS	POS	POS	POS	POS	+	NEG	POS	**
35	FG35	*Aspergillus clavatus*	MG647856	POS	POS	POS	NEG	NEG	NEG	POS	-	nd	nd	
36	FG36	*Aspergillus oryzae*	MG659633	POS	POS	POS	POS	POS	NEG	POS	+	NEG	POS	**
37	FG37	*Aspergillus terreus*	MG647866	POS	POS	POS	NEG	POS	NEG	POS	+	nd	nd	
38	FG38	*Aspergillus terreus*	MG647852	POS	POS	POS	NEG	POS	NEG	POS	-	nd	nd	
39	FG39	*Aspergillus flavus*	MG647857	POS	POS	POS	POS	NEG	NEG	POS	-	nd	nd	
40	FG40	*Aspergillus flavus*	MG659627	POS	NEG	POS	POS	POS	POS	POS	+++	POS	POS	**
41	FG41	*Aspergillus parasiticus*	MG659687	POS	POS	POS	POS	POS	POS	POS	+++	POS	POS	**
42	FG42	*Aspergillus flavus*	MG659676	POS	POS	POS	POS	POS	NEG	NEG	-	POS	POS	**
43	FG43	*Aspergillus nomius*	MH270600	POS	POS	POS	POS	POS	POS	POS	+++	POS	POS	**
44	FG44	*Aspergillus ochraceoroseus*	MH270530	POS	POS	POS	POS	POS	POS	POS	++	POS	POS	**
45	FG45	*Aspergillus flavus*	MG647871	POS	POS	POS	POS	NEG	NEG	POS	-	nd	nd	
46	Control 1	*Aspergillus flavus ATCC*	*259622^TM^*	POS	POS	POS	POS	POS	POS	POS	+++	POS	POS	
47	Control 2	*Saccharomyces cereviasiae*		NEG	NEG	NEG	NEG	NEG	NEG	NEG	-	nd	nd	

Keys: POS = positive amplification/reaction, NEG = negative amplification/reaction, + = fluorescence, - = no fluorescence, +/- = low fluorescence, ++ = mild fluorescence, +++ = high fluorescence, ++++ = very high fluorescence, nd = not detected, ** = selected isolates, TLC = thin-layer chromatography, HPLC = high-performance liquid chromatography, YES = yeast extract sucrose medium, and β-CDNRDCA = β-cyclodextrin neutral red desiccated coconut agar.

**Table 3:** Aflatoxin concentrations (ug/g) of wheat flour inoculated with *Aspergillus* strains isolated from feedlots of animals kept for food production.

S/N	ID	Isolate Name	AFG2 (µg/g)	AFG1 (µg/g)	AFB2 (µg/g)	AFB1 (µg/g)	AFTOT (µg/g)
1	FG4	*Aspergillus flavus*	nd	nd	6.78 ± 0.01 ^r^	nd	6.78 ± 0.01 ^q^
2	FG7	*Aspergillus flavus*	1.67 ± 0.01 ^p^	nd	nd	54.75 ± 3.46 ^f^	56.42 ± 3.45 ^m^
3	FG9	*Aspergillus flavus*	nd	nd	nd	nd	nd
4	FG10	*Aspergillus flavus*	nd	6.21 ± 0.02 ^c^	nd	nd	6.21 ± 0.02 ^q^
5	FG12	*Aspergillus parasiticus*	360.06 ± 0.05 ^b^	745.34 ± 0.03 ^a^	242.52 ± 0.04 ^a^	34,043.71 ± 0.27 ^b^	35,391.63 ± 0.34 ^b^
6	FG14	*Aspergillus flavus*	nd	nd	13.24 ± 0.01 ^q^	59.66 ± 0.02 ^e^	72.90 ± 0.01 ^l^
7	FG15	*Aspergillus nomius*	768.52 ± 0.03 ^a^	396.45 ± 0.02 ^b^	nd	70,289.23 ± 0.67^a^	71,454.21 ± 0.66 ^a^
8	FG18	*Aspergillus flavus*	21.91 ± 0.03 ^i^	nd	103.15 ± 0.02 ^c^	nd	125.06 ± 0.03 ^h^
9	FG19	*Aspergillus flavus*	0.98 ± 0.02 ^q^	nd	24.14 ± 0.01 ^o^	nd	25.12 ± 0.03 ^p^
10	FG20	*Aspergillus flavus*	11.84 ± 0.04 ^k^	nd	66.08 ± 0.07 ^i^	nd	77.92 ± 0.11 ^j^
11	FG22	*Aspergillus flavus*	3.43 ± 0.02 ^o^	1.88 ± 0.01 ^f^	50.08 ± 0.01 ^k^	nd	55.39 ± 0.04 ^m^
12	FG23	*Aspergillus flavus*	72.55 ± 0.02 ^e^	nd	30.33 ± 0.04 ^m^	nd	102.88 ± 0.04 ^i^
13	FG24	*Aspergillus flavus*	14.86 ± 0.06 ^j^	nd	57.77 ± 0.02 ^j^	nd	72.63 ± 0.06 ^l^
14	FG25	*Aspergillus oryzae*	86.04 ± 0.04 ^d^	nd	95.58 ± 0.01 ^d^	nd	181.62 ± 0.05 ^f^
15	FG31	*Aspergillus flavus*	253.44 ± 0.05 ^c^	3.19 ± 0.02 ^d^	73.88 ± 0.03 ^f^	nd	330.51 ± 0.07 ^e^
16	FG33	*Aspergillus flavus*	38.33 ± 0.03 ^g^	nd	109.23 ± 0.03 ^a^	nd	147.57 ± 0.05 ^g^
17	FG34	*Aspergillus oryzae*	nd	nd	nd	nd	nd
18	FG36	*Aspergillus oryzae*	nd	nd	nd	nd	nd
19	FG40	*Aspergillus flavus*	4.77 ± 0.02 ^m^	nd	71.66 ± 0.02 ^h^	nd	76.43 ± 0.03 ^k^
20	FG41	*Aspergillus parasiticus*	29.44 ± 0.04 ^h^	nd	45.09 ± 0.01 ^l^	1062.56 ± 0.05 ^c^	1137.09 ± 0.08 ^c^
21	FG42	*Aspergillus flavus*	4.65 ± 0.03 ^n^	nd	73.73 ± 0.02 ^g^	nd	78.39 ± 0.06 ^j^
22	FG43	*Aspergillus nomius*	56.57 ± 0.03 ^f^	0.84 ± 0.01 ^g^	75.63 ± 0.02 ^e^	704.67 ± 0.02 ^d^	837.70 ± 0.06 ^d^
23	FG44	*Aspergillus ochraceoroseus*	11.47 ± 0.02 ^l^	2.34 ± 0.02 ^e^	22.65 ± 0.02 ^p^	3.77 ± 0.02 ^g^	40.23 ± 0.05 ^n^
24	Control 1	*Aspergillus flavus ATCC* 46283	0.53 ± 0.07 ^s^	nd	29.35 ± 0.03 ^n^	nd	29.87 ± 0.05 ^o^
25	Control 2	*Saccharomyces cerevisiae*	0.63 ± 0.03 ^r^	nd	0.04 ± 0.01 ^s^	nd	0.67 ± 0.03
26	Standard	AFG2 (0.05) µg/mL	13,820.28	nd	nd	nd	nd
27	standard	AFG1 (0.05) µg/mL	nd	4,556,586	nd	nd	nd
28	standard	AFB2 (0.05) µg/mL	nd	nd	1,798,698	nd	nd
29	standard	AFB1 (0.05) µg/mL	nd	nd	nd	3,742,170	nd
30	standard	AFTOT (0.05) µg/mL	nd	nd	nd	nd	11,479,481
	Standard	WHO/FAO limit in Feed [26]					5 µg/kg
		European Union limit in animal feed [27]					4 µg/kg
		European Commission aflatoxins in animal feed [14,28]					5–20 µg/kg
		European Commission tolerable limit of aflatoxins in foods in Africa [28]					<5–20 µg/kg
		European Commission tolerable limits of aflatoxins in Animal feeds [28]					<0.001–0.01 µg/kg body weight
		European Commission aflatoxins limits for infants food [28]					0.05–10 µg/kg

Keys; FG = fungal isolates description; AFB1, AFB2, AFG1, AFG2 = Aflatoxins; and AFTOT = total aflatoxin. Superscript are significantly different across the column at *p* ≤ 0.05.

## Supplementary Materials

The following are available online at https://www.mdpi.com/2072-6651/11/12/692/s1, Table S1: PCR Conditions for Amplification of Aflatoxin Biosynthesis Genes.
