# Supplementary material for: Polyphasic Assessment of Aflatoxin Production Potential in Selected Aspergilli
Source: Toxins (Basel). 2019 Nov 26;11(12):692. doi: 10.3390/toxins11120692 (PMC6950480; doi:10.3390/toxins11120692)
Supplement: Supplementary file 1 [file toxins-11-00692-s001.pdf]

Supplementary Materials: Polyphasic Assessment of Aflatoxin Production Potential in Selected *Aspergilli*

Stephen Abiola Akinola, Collins Njie Ateba and Mulunda Mwanza

Table S1. PCR Conditions for Amplification of Aflatoxin Biosynthesis Genes.

S/N	Target Gene	Primer sets (5′–3′) Forward	Primer sets (5′–3′) Reverse	PCR Process	Conditions	Amplicon Size
**1**	*AflR*	TAT CTC CCC CCG GGC ATC TCC CGG	CCG TCA GAC AGC CAC TGG ACA CGG	Pre-denaturation	95 °C, 4 min	1032 bp
				Denaturation	95 °C, 1 min	
				Annealing	60 °C, 1 min	
				Extension (cycles)	72 °C, 30 s (30 cycles)	
				Final elongation	72 °C, 10 min (1 cycle)	
				Hold temperature	4 °C	
**2**	*afl*J	TGA ATC CGT ACC CTT TGA GG-	GGA ATG GGA TGG AGA TGA GA	Pre-denaturation	95 °C, 10 min	737 bp
				Denaturation	95 °C, 50 s	
				Annealing	58 °C, 50 s	
				Extension (cycles)	72 °C, 2 min (30cycles)	
				Final elongation	72 °C, 10 min (1 cycle)	
				Hold temperature	4 °C	
**3**	*afl*D (Nor)	ACC GCT ACG CCG GCA CTC TCG GCA C	GTT GGC CGC CAG CTT CGA CAC TCC G	Pre-denaturation	94 °C, 10 min	400 bp
				Denaturation	94 °C, 1 min	
				Annealing	65 °C, 1 min	
				Extension (cycles)	72 °C, 2 min (33cycles)	
				Final elongation	72 °C, 5 min (1 cycle)	
				Hold temperature	4 °C	
**4**	*afl*M	GCC GCA GGC CGC GGA GAA AGT GGT	GGG GAT ATA CTC CCG CGA CAC AGC C	Pre-denaturation	95 °C, 4 mins	537 bp
				Denaturation	95 °C, 1 min	
				Annealing	58 °C, 1 min	

				Extension (cycles)	72 °C, 30 s (30 cycles)	
				Final elongation	72 °C, 10 mins (1 cycle)	
				Hold temperature	4 °C	
**5**	Omt-A	GTG GAC GGA CCT AGT CCG ACA TCA C	GTC GGC GCC ACG CAC TGG GTT GGG G	Pre-denaturation	94 °C, 5 min	797 bp
				Denaturation	94 °C, 1 min	
				Annealing	75 °C, 2 mins	
				Extension (cycles)	72 °C, 2 min (33cycles)	
				Final elongation	10 min (1 cycle)	
				Hold temperature	4 °C	

Adapated from Adetunji *et al.* (2019).
